# Clinical and epidemiological characteristics of cystic echinococcosis in children from a tertiary center in Peru

**DOI:** 10.17843/rpmesp.2022.391.9830

**Published:** 2022-03-31

**Authors:** Blanca Salazar-Mesones, Medalit Luna-Vílchez, Julio Maquera-Afaray, Christian Chiara-Chilet, Diana Portillo-Álvarez, José W. López

**Affiliations:** 1 Instituto Nacional de Salud del Niño San Borja, Lima, Peru. Instituto Nacional de Salud del Niño San Borja Lima Peru; 2 Universidad Privada de Tacna, Tacna, Peru. Universidad Privada del Norte Universidad Privada de Tacna Tacna Peru; 3 Universidad Científica del Sur, Lima, Peru. Universidad Científica del Sur Universidad Científica del Sur Lima Peru

**Keywords:** Echinococcosis, *Echinococcus granulosus*, Echinococcosis, Hepatic, Echinococcosis, Pulmonary, Children

## Abstract

Cystic echinococcosis (CE) in children is a public health problem. To describe the clinical and epidemiological profile of CE, we reviewed the records of 55 children admitted to our institution with a confirmed diagnosis of CE between 2017-2019, analyzing demographic data, clinical manifestations, and treatment. Of the population, 61.8% (34/55) were male. The mean age was 9.25 years (SD: 2.79); 16.4% had previous CE diagnosis, and 50.9% had contact with dogs. The median time of illness was 2 months. Of the patients, 65.5% had hepatic involvement, 56.4% had pulmonary involvement, and 21.8% had both hepatic and pulmonary involvement. The most frequent symptoms were abdominal pain (80.6%) and cough (80.6%). Surgical treatment was performed in 87.5% of patients with hepatic CE, in 100% of those with pulmonary CE and in 100% of those with hepatic and pulmonary CE. Albendazole was prescribed in 100% of hepatic cases, in 73.7% of pulmonary cases, and in 75% of those with both conditions. Mortality was not reported.

## INTRODUCTION

Cystic echinococcosis (CE) is a parasitic disease caused by *Echinococcus granulosus sensu lato* (*sl*) which produces chronic, often silent, infections in humans ^1^. *Echinococcus spp* causes the following diseases: cystic echinococcosis (CE), alveolar echinococcosis (AE) and neotropical echinococcosis (NE) ^2^.

Parasites of the species *E. granulosus sensu stricto* (genotypes G1/G3) cause the majority of human infections (88%) ^3^. *E. granulosus* is distributed worldwide, but is frequently found in South America, Africa and Asia; and endemic in Argentina, Brazil, Uruguay, Chile and Peru, countries with important livestock and grazing activity ^4^.

*E. granulosus* sl. requires two mammalian hosts to complete its life cycle; the adult tapeworm is found in the small intestine of dogs and other canids, while the larval stage is located in the viscera of ungulates, especially sheep and goats ^5^. Transmission occurs between definitive hosts (canids) and intermediate hosts (herbivores). Dogs play an important role in the life cycle of *E. granulosus* sl. because they can contaminate the environment with parasite eggs, which can remain viable for a long time^ (^ ^6^ ^,^ ^7^. Thus, man becomes an accidental host by coming into direct contact with parasitized dogs or by ingesting parasite eggs ^8^.

In 2012, the Food and Agriculture Organization of the United Nations (FAO) and the World Health Organization (WHO) ranked *E. granulosus* second among foodborne parasites of global public health importance ^9^. Most cases are diagnosed in adults because of the slow growth of the parasite, and only 10-20% of cases are diagnosed in children under 16 years of age ^10^. The existence of pediatric cases suggests an active community transmission cycle ^11^. This zoonosis is an important public health problem in several countries and an emerging and re-emerging disease in others ^10^.

The aim of the study was to describe the clinical and epidemiological characteristics of pediatric patients with CE in a referral surgical center in Peru.

**KEY MESSAGES**

Motivation for the study: Cystic echinococcosis (CE) is a public health problem in Peru. Information on pediatric cases is scarce. The Instituto Nacional de Salud del Niño San Borja is a pediatric surgical referral center.

Main findings: Hepatic affection was the most frequent form and few patients completed follow-up because they came from the provinces. No mortality was found.

Implications: The findings of our study could be useful to improve the referral processes in children with CE in need of surgery.

## THE STUDY

### Design and study population

Retrospective study in patients under 18 years of age with confirmed diagnosis of CE, attended at the Instituto Nacional de Salud del Niño San Borja (INSN-SB), in Lima, between 2017 and 2019. We excluded patients with incomplete data in the clinical record regarding the analyzed variables.

### Study variables

The studied epidemiologic variables were sex, age, place of origin, previous diagnosis of CE in patients and family members, and contact with dogs. The clinical variables were time of illness measured from symptom onset to contact with a healthcare center and symptoms such as cough, hemoptysis, dyspnea, chest pain, abdominal pain, vomiting, nausea, fever, decreased weight, and decreased appetite.

Confirmatory diagnosis of CE was made by histopathological study of the surgical and/or serological specimen, with Western blot and/or IgG ELISA. We characterized the cysts according to the WHO-IWGE ^12^ echotomographic classification, which monitors the natural history, from a simple cyst to a transitional stage and ends with its inactivation. The treatment variables were the type of therapy, dosage, frequency and duration. Finally, hospital stay was evaluated considering the final status of the patient.

### Data analysis

Stata® v16 statistical software (Stata Corporation, College Station, Texas, USA) was used for the analysis. Descriptive statistics were used, quantitative variables were represented by measures of central tendency and dispersion according to their normality distribution, while qualitative variables were summarized using frequencies and percentages.

## FINDINGS

We identified 55 patients under 18 years of age with CE diagnosis; 61.8% (34/55) were male. The mean age was 9.25 years (SD: 2.79) and the greatest number of cases (67.3%, 37/55) were between 6 and 11 years old. From the total of patients, 81.8% (45/55) were from the provinces, with Pasco being the department with the highest number of cases (21.8%, 12/55). Cases reported in the department of Lima accounted for 18.2% (10/55) (Table 1). Contact with dogs was observed in 50.9% (28/55) of the patients; previous CE diagnosis in patients and in their relatives was 16.4% (9/55) and 3.6% (2/55), respectively. Most patients presented symptoms (94.5%, 52/55), and only three were diagnosed incidentally. The median time of illness was 2 months (IQR: 1-4). Liver involvement was found in 65.5% (36/55) of the patients, 56.4% (31/55) showed pulmonary involvement, while 21.8% (12/55) had both hepatic and pulmonary involvement.

**Table 1:** Demographic characteristics of patients diagnosed with cystic echinococcosis from the Instituto Nacional de Salud del Niño San Borja (n=55)

Characteristics	Total	
	n	%
Sex	34	61.8
Male	21	38.2
Female		
Age		
1 to 5 years	7	12.7
6 to 11 years	37	67.3
12 to 18 years	11	20.0
Place of origin		
Pasco	12	21.8
Junín	11	20.0
Lima	10	18.2
Huancavelica	10	18.2
Ayacucho	7	12.8
Ancash	1	1.8
Cusco	1	1.8
Puno	1	1.8
Lambayeque	1	1.8
Piura	1	1.8

In cases of hepatic CE, the most frequent symptom was abdominal pain (80.6%, 29/36); 75.0% (27/36) had cysts larger than 5 cm and 33.3% (12/36) had more than one lesion. In cases of pulmonary CE, the most frequent symptom was cough (80.6%, 25/31); 67.7% (21/31) had single cyst and 61.3% (19/31) had cysts larger than 5 cm (Table 2).

**Table 2:** Clinical characteristics of patients with a diagnosis of cystic echinococcosis seen at the Instituto Nacional de Salud del Niño San Borja (n=55).

Characteristics	Total	
	n	%
Location		
Liver	24	43.6
Lung	19	34.6
Hepatopulmonary	12	21.8
Number of hepatic lesions		
One	24	66.7
Two or more	12	33.3
Number of Pulmonary lesions		
One	21	67.7
Two or more	10	32.2
Signs and symptoms		
Hepatic involvement		
Abdominal pain	29	80.6
Fever	5	13.9
Vomiting	5	13.9
Nausea	4	11.1
Jaundice	2	5.6
Decreased weight	2	5.6
Pulmonary involvement		
Cough	25	80.6
Hemoptysis	15	48.4
Dyspnea	10	32.3
Fever	10	32.3
Chest pain	6	19.4

According to the WHO-IWGE classification ^12^, we found a higher percentage of cysts in early stages (CE1) (52.8%, 19/36). Tomography (56.4%, 31/55) was the most frequent diagnostic method, and 74.5% (41/55) of the cases were confirmed by pathological anatomy (Table 3). Among the patients with CE serology, 37.5% (6/16) had positive IgG Elisa, and 76.9% (10/13) of those who underwent Western blot for CE (13/16) were positive.

**Table 3:** Diagnostic characteristics of patients with cystic echinococcosis from the Instituto Nacional de Salud del Niño San Borja (n=55).

Characteristics	Total	
	n	%
WHO-IWGE classification		
CE1	19	52.8
CE2	2	5.6
CE3	11	30.6
CE4	2	5.6
CE5	1	2.8
Diagnostic		
Serologic	16	29.1
Radiologic	15	27.3
Ultrasound	16	29.1
Tomographic	31	56.4
Anatomopathological	41	74.5

CE1: unilocular lesion with visible cystic wall. CE2: multivesicular lesion, visible daughter vesicles. CE3: unilocular lesion, detachment of the lamellar membrane within the cyst. CE4: heterogeneous hypo- or hyperechogenic lesion, with degenerative content. CE5: calcification of the cystic wall, total or partial.

Surgery was needed in 87.5% (21/24) of the cases of hepatic CE; of these, 76.2% (16/24) had pre-surgical albendazole prophylaxis, and 100% (21/21) received albendazole post-surgery for four weeks of treatment, on average. Those cases that only received albendazole (12.5%, 3/24) had cysts smaller than 4 cm.

All pulmonary CE cases (100%, 19/19) were surgically treated; of these, 26.3% (5/19) received pre-surgical prophylaxis with albendazole for ruptured cysts or fissures (Table 4). The dose used for albendazole was 7.5 mg/kg/dose every 12 h (maximum 400 mg every 12 h). Of all the cases, 9.1% (5/55) had new lesions after treatment; 60.0% (3/5) after 12 months and the other two cases at 7 and 8 months. The median number of days of hospitalization was 9 (IQR: 7-16). No mortality was reported.

**Table 4:** Therapeutic characteristics of patients diagnosed with cystic echinococcosis from the Instituto Nacional de Salud del Niño San Borja (n=55).

Characteristic	Hepatic CE (n=24)		Pulmonary CE (n=19)		Hepatopulmonary CE (n=12)	
	n	%	n	%	n	%
Surgical treatment	21	87.5	19	100	12	100
Pre-surgical deworming prophylaxis	16	76.2	5	26.3	6	50
Post-surgery antiparasitic treatment	21	100	14	73.7	9	75
Only antiparasitic treatment	3	12.5	-	-	-	-

## DISCUSSION

Most CE cases treated at INSN-SB were patients between 6 and 11 years of age with hepatic involvement, who received surgical and medical treatment, with no mortality. This profile is similar to that published by Huamán G. *et al*. ^13^ and Stiglich *et al*. ^14^ who reported a higher percentage in children between 5 and 9 years of age (50.8% and 64.0%, respectively). This implies contact with the parasite at an early age, given that cystic growth is approximately 1 cm per year ^15^. In our study we found a low proportion of family members with previous CE diagnosis, probably due to the lack of a search for intrafamilial cases at the local level.

From 2009 to 2014, 29,559 new human CE cases of were reported in Argentina, Brazil, Chile, Peru, and Uruguay, with approximately 880 deaths reported in the five countries; the proportion of cases in children under 15 years of age was 15%, showing active community transmission to children ^16^. Departments located in the central highlands reported the highest number of cases. These regions have intense livestock and grazing activity and poor sanitary conditions, which would favor fecal-oral transmission with dog feces through close and playful contact ^10^.

Several studies report that hepatic involvement in CE is frequent; our casuistry also shows a higher percentage of cases with hepatic involvement; a retrospective study in Spain ^17^ showed that 55% of children under 15 years of age had hepatic involvement. In contrast, Huamán G. *et al*. ^13^ found a greater pulmonary involvement (44%) compared to hepatic (23.2%). 

Clinical manifestations are diverse and depend on the site, size and state of the cyst. Hepatic CE may be asymptomatic until the cyst reaches a certain size. We found that abdominal pain is the most frequent symptom in hepatic CE ^18^ and cough is the most frequent one in pulmonary CE, which is similar to what was reported by Huamán *et al* ^13^. Most primary infections in humans consist of a single cyst ^10^, as found in our study for both hepatic and pulmonary CE.

Techniques such as diagnostic imaging or immunoassays are reported as diagnostic tools. Abdominal ultrasound is an important technique for hepatic CE because of its availability and usefulness in defining dimensions, number, site and status of cysts. Computed tomography can provide additional information when cysts are not visible during ultrasound. In our study, computed tomography was the most commonly employed method, while serology was used in a smaller proportion.

Although antibody testing is useful when confirming the diagnosis, not all patients with CE have a detectable immune response ^10^. The sensitivity of serological tests varies between 75 to 80% and is inversely related to the degree of antigens sequestration within the cyst; thus, intact cysts elicit minimal immune response, in contrast, fissured or ruptured cysts exhibit greater immune response ^10^.

False negative results may be related to early (CE1) or late (CE5) stages of the disease. The Western blot assay is used as a confirmatory test due to its higher sensitivity and specificity; it is also reported that antibody titers decrease after surgical treatment, not being able to discriminate between active and past infections ^19^. In our study, 76.9% (10/13) of the patients had positive serology for CE Western blot.

The preferred treatment for CE is surgical, and complete excision of the cyst without leakage leads to cure ^10^. In the case of unresectable cysts or cysts smaller than 5 cm, benzimidazole derivatives such as albendazole have shown to be effective as an alternative or complementary therapy to surgery, showing a significant regression of the cyst size and relief of symptoms ^10^ ^,^ ^12^. In our study we found a higher percentage of surgical treatment for both cases. Patients were treated with albendazole at a dose of 7.5 mg/kg/dose every 12 h orally (maximum 400 mg every 12 h). Albendazole is the most commonly used drug in children with CE, with limited experience in the treatment of children under 6 years of age. Although in the past albendazole was used 14 days per month for 3 months, it is currently prescribed for 3 to 6 consecutive months ^20^; however, few studies are available on the indication and length of treatment in children.

Few cases have recurrence after treatment; therefore, it is important to follow-up using diagnostic imaging every 3 months (ultrasound or tomography) for a minimum of 3 years ^10^. Likewise, it is recommended to screen the patient’s relatives in order to have a timely and early diagnosis of possible new cases.

In our study, hospital stay was short and showed adequate postoperative evolution. Few cases had complicated CE (complicated liver cyst and cholangitis; complicated liver cyst with more than ten lesions, liver cyst plus intra-abdominal abscess and pulmonary cyst plus empyema). No mortality was reported.

An active epidemiological surveillance program in endemic areas is also necessary to control the disease. Early CE diagnosis in asymptomatic children will be achieved as long as access to imaging and serologic tests improves

Our study had limitations, such as the lack of standardization of the clinical information from the medical records, the correct classification and detailed description of the cysts, time of albendazole therapy and the criteria for the prescription of antiparasitic prophylaxis. Since INSN-SB receives patients referred with previous laboratory results from various centers and laboratories in the country, the information from these auxiliary tests was included for surgical decision making in each case.

In conclusion, CE was more frequent in school-age children, the liver was the most affected organ, most patients were surgically treated and no fatal events were reported.
