# Supplementary material for: Clinical and epidemiological characteristics of cystic echinococcosis in children from a tertiary center in Peru
Source: Rev Peru Med Exp Salud Publica. 2022 Mar 31;39(1):65–9. doi: 10.17843/rpmesp.2022.391.9830 (PMC11397754; doi:10.17843/rpmesp.2022.391.9830)
Supplement: Translation

# Translation: Características clínicas y epidemiológicas de equinococosis quística en niños de un centro terciario en Perú

**Authors:** Blanca Salazar-Mesones, Medalit Luna-Vílchez, Julio Maquera-Afaray, Christian Chiara-Chilet, Diana Portillo-Álvarez, José W. López

**Affiliations:** 1 Instituto Nacional de Salud del Niño San Borja, Lima, Perú.; 2 Universidad Privada de Tacna, Tacna, Perú.; 3 Universidad Científica del Sur, Lima, Perú.

**Keywords:** Equinococosis Quística, *Echinococcus granulosus*, Equinococosis Hepática, Equinococosis Pulmonar, Niños

## Abstract

La equinococosis quística (EQ) en niños es un problema de salud pública. Para describir las características clínicas y epidemiológicas de la EQ se revisaron los registros de 55 niños con diagnóstico confirmado de EQ admitidos entre 2017 y 2019 en un centro quirúrgico referencial del Perú. Se analizaron los datos demográficos, las manifestaciones clínicas y el tratamiento. El 61,8% (34/55) de los niños fue de sexo masculino. La edad promedio fue de 9,25 años (DE: 2,79); un 16,4% tuvo diagnóstico previo de EQ, y un 50,9% tuvo contacto con perros. La mediana de tiempo de enfermedad fue de dos meses. El 65,5% tuvo afectación hepática, el 56,4% pulmonar y el 21,8% hepática y pulmonar. Los síntomas más frecuentes fueron dolor abdominal (80,6%) y tos (80,6%). El tratamiento quirúrgico se realizó en el 87,5% de los casos con EQ hepática y en el 100% de los casos con EQ pulmonar y EQ hepática y pulmonar. Se prescribió albendazol en el 100% de casos hepáticos, en el 73,7% de casos pulmonares y en el 75% de ambas afectaciones. No se reporta mortalidad.

## INTRODUCCIÓN

La equinococosis quística (EQ) es una enfermedad parasitaria,* Echinococcus granulosus sensu lato (sl)* genera infecciones crónicas en humanos, muchas veces silentes ^1^. Las formas de enfermedad producidas por *Echinococcus spp *son: la equinococosis quística (EQ), la equinococosis alveolar (EA) y la equinococosis neotropical (EN) ^2^. 

Los parásitos de la especie *E. granulosus sensu stricto* (genotipos G1/G3) causan la mayoría de las infecciones humanas (88%) ^3^. La EQ tiene una distribución mundial, y es frecuente, en Sudamérica, África y Asia; y endémica en Argentina, Brasil, Uruguay, Chile y Perú, países con importante actividad ganadera y de pastoreo ^4^.

*E. granulosus sl.* requiere dos huéspedes mamíferos para completar su ciclo de vida; la tenia adulta se encuentra en el intestino delgado de los perros y otros cánidos, mientras que la etapa larvaria se localiza en las vísceras de los ungulados, especialmente ovejas y cabras ^5^. La transmisión ocurre entre huéspedes definitivos (cánidos) y huéspedes intermediarios (herbívoros). Los perros tienen un papel importante en el ciclo de vida de *E. granulosus sl.* porque pueden contaminar el medioambiente con huevos de parásitos, los cuales pueden permanecer viables durante mucho tiempo ^6^ ^,^ ^7^. Así, el hombre se convierte en huésped accidental al ponerse en contacto directo con perros parasitados o por la ingestión de los huevos de parásitos ^8^.

En 2012, la Organización de las Naciones Unidas para la Agricultura y la Alimentación (FAO) y la Organización Mundial de la Salud (OMS) clasificaron al *E. granulosus* en segundo lugar entre los parásitos transmitidos por alimentos de importancia para la salud pública mundial ^9^. La mayoría de los casos se diagnostican en adultos por la lentitud de crecimiento del parásito, y solo el 10-20% de casos se diagnostican en menores de 16 años ^10^. La presencia de casos pediátricos sugiere un ciclo de transmisión comunitario activo ^11^. Esta zoonosis es un importante problema de salud pública en varios países y en otros constituye una enfermedad emergente y reemergente ^10^.

El objetivo del estudio fue describir las características clínicas y epidemiológicas de pacientes pediátricos con EQ en un centro quirúrgico referencial del Perú.

**MENSAJES CLAVE**

Motivación para realizar el estudio: la equinococosis quística (EQ) es un problema de salud pública en Perú. La información de afectación pediátrica es escasa. El Instituto Nacional de Salud del Niño San Borja es un centro referencial quirúrgico pediátrico.

Principales hallazgos: la forma mas frecuente fue la afectación hepática, pocos pacientes completaron el seguimiento por provenir de provincias. No se halló mortalidad.

Implicancias: los hallazgos de este estudio podrían servir para mejorar los procesos de referencia en los niños con EQ con indicación de cirugía.

## EL ESTUDIO

### Diseño y población de estudio

Estudio retrospectivo en pacientes menores de 18 años con diagnóstico confirmado de EQ, atendidos en el Instituto Nacional de Salud del Niño San Borja (INSN-SB), en Lima, entre el 2017 y 2019. Se excluyeron pacientes con datos incompletos en la historia clínica para las variables analizadas. 

### Variables de estudio

Las variables epidemiológicas estudiadas fueron sexo, edad, procedencia, diagnóstico previo de EQ en pacientes y familiares, y contacto con perros. Las variables clínicas incluidas fueron el tiempo de enfermedad desde el inicio de síntomas hasta el contacto con un centro de salud y síntomas como tos, hemoptisis, disnea, dolor torácico, dolor abdominal, vómitos, náuseas, fiebre, disminución ponderal e hiporexia.

El diagnóstico confirmatorio de EQ se realizó mediante el estudio histopatológico de la muestra quirúrgica y/o serológica, con Western blot y/o ELISA IgG. Para caracterizar el quiste de EQ se usó la clasificación ecotomográfica de OMS-IWGE ^12^ que monitorea la historia natural, desde el paso de un quiste simple a una etapa de transición y finaliza con su inactivación. Las variables de tratamiento consideradas fueron el tipo de terapia, dosificación, frecuencia y duración. Finalmente, se evaluó la estancia hospitalaria considerando la condición final del paciente. 

### Análisis de datos

Para el análisis se utilizó el *software* estadístico Stata® v16 (Stata Corporation, College Station, Texas, USA). Se empleó estadística descriptiva, donde las variables cuantitativas fueron representadas por medidas de tendencia central y de dispersión según su distribución de normalidad, mientras que las variables cualitativas se resumen usando frecuencias y porcentajes.

### Aspectos éticos

El estudio fue aprobado por el Comité de Ética en Investigación del INSN-SB, bajo el código institucional PI-305-2019.

## HALLAZGOS

Se identificaron 55 pacientes menores de 18 años con diagnóstico de EQ. El 61,8% (34/55) fueron varones. La media de edad fue de 9,25 años (DE: 2,79) y el mayor número de casos (67,3%, 37/55) tuvieron entre 6 y 11 años. El 81,8% (45/55) procedían de provincia, siendo Pasco el departamento con mayor número de casos (21,8%, 12/55). Los casos reportados en el departamento de Lima representaron el 18,2% (10/55) (Tabla 1). El contacto con perros se observó en el 50,9% (28/55), mientras que el diagnóstico previo de EQ en los pacientes fue de 16,4% (9/55) y en familiares fue de 3,6% (2/55). La mayoría de los pacientes presentaron síntomas (94,5%, 52/55), y solo tres fueron diagnosticados incidentalmente. La mediana del tiempo de enfermedad fue 2 meses (RIC: 1-4). La afectación fue hepática en el 65,5% (36/55) y pulmonar en el 56,4% (31/55), mientras que el 21,8% (12/55) tuvo afectación hepática y pulmonar.

**Tabla 1:** Características demográficas de los pacientes con diagnóstico de equinococosis quística atendidos en el Instituto Nacional de Salud del Niño San Borja (n=55).

Característica	Total	
	n	%
Sexo		
Masculino	34	61,8
Femenino	21	38,2
Edad		
1 - 5 años	7	12,7
6 - 11 años	37	67,3
12 - 18 años	11	20,0
Procedencia		
Pasco	12	21,8
Junín	11	20,0
Lima	10	18,2
Huancavelica	10	18,2
Ayacucho	7	12,8
Ancash	1	1,8
Cusco	1	1,8
Puno	1	1,8
Lambayeque	1	1,8
Piura	1	1,8

En los casos de EQ hepática, el síntoma más frecuente fue el dolor abdominal (80,6%, 29/36); el 75,0% (27/36) tuvo quistes mayores de 5 cm y el 33,3% (12/36) presentó más de una lesión. En los casos de EQ pulmonar, el síntoma más frecuente fue la tos (80,6%, 25/31); el 67,7% (21/31) tuvo quiste único y el 61,3% (19/31) presentó quistes mayores de 5 cm (Tabla 2).

**Tabla 2:** Características clínicas de los pacientes con diagnóstico de equinococosis quística atendidos en el Instituto Nacional de Salud del Niño San Borja (n=55).

Características	Total	
	n	%
Localización		
Hepática	24	43,6
Pulmonar	19	34,6
Hepatopulmonar	12	21,8
Número de lesiones hepáticas		
Uno	24	66,7
Dos o más	12	33,3
Número de lesiones pulmonares		
Uno	21	67,7
Dos o más	10	32,3
Signos y síntomas		
Afectación hepática		
Dolor abdominal	29	80,6
Fiebre	5	13,9
Vómito	5	13,9
Náuseas	4	11,1
Ictericia	2	5,6
Hiporexia	2	5,6
Afectación pulmonar		
Tos	25	80,6
Hemoptisis	15	48,4
Disnea	10	32,3
Fiebre	10	32,3
Dolor torácico	6	19,4

Según la clasificación de OMS-IWGE ^12^ se encontró un mayor porcentaje de quistes en etapas tempranas (CE1) (52,8%, 19/36). El método diagnóstico más frecuente fue la tomografía con el 56,4% (31/55), y el 74,5% (41/55) de los casos se confirmó mediante anatomía patológica (Tabla 3). Entre los pacientes con serología para la EQ, el 37,5% (6/16) contó con Elisa IgG positivo, y en los que se realizaron Western blot para EQ (13/16) resultaron positivos el 76,9% (10/13).

**Tabla 3:** Características diagnósticas de los pacientes con equinococosis quística atendidos en el Instituto Nacional de Salud del Niño San Borja (n=55).

Características	Total	
	n	%
Clasificación OMS-IWGE		
CE1	19	52,8
CE2	2	5,6
CE3	11	30,6
CE4	2	5,6
CE5	1	2,8
Diagnóstico		
Serológico	16	29,1
Radiológico	15	27,3
Ecográfico	16	29,1
Tomográfico	31	56,4
Anatomopatológico	41	74,5

CE1: lesión unilocular con pared quística visible. CE2: lesión multivesicular, vesículas hijas visibles. CE3: lesión unilocular, desprendimiento de la membrana laminar dentro del quiste. CE4: lesión heterogénea hipo o hiperecogénica, con contenido degenerativo. CE5: calcificación de la pared quística, total o parcial.

El tratamiento fue quirúrgico en el 87,5% (21/24) de los casos de EQ hepática; de ellos, el 76,2% (16/24) tuvo profilaxis prequirúrgica con albendazol, y el 100% (21/21) recibió albendazol poscirugía por cuatro semanas de tratamiento, en promedio. Los casos que solo recibieron albendazol (12,5%, 3/24) tuvieron quistes menores de 4 cm. 

Todos los casos de EQ pulmonar (100%,19/19) tuvieron tratamiento quirúrgico; de ellos, el 26,3% (5/19) recibió profilaxis prequirúrgica con albendazol por quistes rotos o fisurados (Tabla 4). La dosis utilizada para el albendazol fue de 7,5 mg/kg/dosis cada 12 h (máximo 400 mg cada 12 h). El 9,1% (5/55) presentó nuevas lesiones después del tratamiento; de ellos, el 60,0% (3/5) después de 12 meses y los otros dos casos a los 7 y 8 meses. La mediana de los días de hospitalización fue de 9 días (RIC: 7-16). No se reportó mortalidad.

**Tabla 4:** Características terapéuticas de los pacientes con diagnóstico de equinococosis quística atendidos en el Instituto Nacional de Salud del Niño San Borja (n=55).

Característica	EQ hepática (n=24)		EQ pulmonar (n=19)		EQ hepática y pulmonar (n=12)	
	n	%	n	%	n	%
Tratamiento quirúrgico	21	87,5	19	100	12	100
Profilaxis antiparasitaria prequirúrgica	16	76,2	5	26,3	6	50
Tratamiento antiparasitario poscirugía	21	100	14	73,7	9	75
Solo tratamiento antiparasitario	3	12,5	-	-	-	-

## DISCUSIÓN

La mayoría de los casos de EQ atendidos en el INSN-SB fueron pacientes entre 6 y 11 años y con afectación hepática, quienes recibieron tratamiento quirúrgico y médico, sin mortalidad. Este perfil es similar a lo publicado por Huamán G. *et al.* ^13^ y Stiglich *et al.* ^14^ quienes reportaron un mayor porcentaje en niños entre 5 y 9 años (50,8 y 64,0%, respectivamente). Esto supone contacto con el parásito en edades tempranas, dado que el crecimiento quístico es de 1cm por año, aproximadamente ^15^. En nuestro estudio se observa baja proporción de familiares con diagnóstico previo de EQ, debido, probablemente, a la falta de búsqueda de casos intrafamiliares a nivel local.

Del 2009 al 2014 se registraron 29 559 nuevos casos humanos de EQ en Argentina, Brasil, Chile, Perú y Uruguay, con reporte de aproximadamente 880 muertes en los cinco países; la proporción de casos en menores de 15 años fue de 15%, mostrando transmisión comunitaria activa hacia los niños ^16^. Los departamentos de la sierra central refirieron mayor número de casos. Estas regiones cuentan con intensa actividad ganadera y pastoreo, y poseen condiciones sanitarias deficientes, lo cual favorecería la transmisión fecal-oral con heces de perros a través del contacto cercano y lúdico ^10^.

Diversos estudios refieren que la afectación hepática en EQ es frecuente, nuestra casuística muestra también un mayor porcentaje de casos con afectación hepática; un estudio retrospectivo en España^ (^ ^17^ mostró que los niños menores de 15 años tuvieron afectación hepática en el 55%. Contrariamente, Huamán G. *et al*. ^13^, encontraron una mayor afectación pulmonar (44%) frente a la hepática (23,2%).

Las manifestaciones clínicas son variables y dependen del sitio, tamaño y estado del quiste. La EQ hepática puede ser asintomática hasta que el quiste alcanza un tamaño considerable. El síntoma más frecuente en la EQ hepática es el dolor abdominal ^18^ tal como se encontró en nuestro estudio, y en la EQ pulmonar fue la tos, datos similares a lo reportado por Huamán *et al*. ^13^. La mayoría de las infecciones primarias en humanos consiste en un solo quiste ^10^, conforme a lo encontrado en nuestro estudio tanto para la EQ hepática y pulmonar. 

Para el diagnóstico se describen diversas técnicas como estudios de imágenes o inmunoanálisis. La ecografía abdominal es una técnica importante para la EQ hepática dada su disponibilidad y utilidad para definir dimensiones, número, sitio y estado de los quistes. La tomografía computarizada puede aportar información adicional cuando los quistes no son visibles con ecografía. En nuestro estudio la tomografía fue el método más empleado, mientras que la serología fue indicada en menor proporción. 

Aunque el análisis de anticuerpos es útil para confirmar el diagnóstico, no todos los pacientes con EQ muestran una respuesta inmunitaria detectable ^10^. La sensibilidad de las pruebas serológicas varía entre 75 a 80% y está inversamente relacionada con el grado de secuestro de los antígenos dentro del quiste; así, los quistes intactos provocan una mínima respuesta inmune, en contraste, los quistes fisurados o rotos presentan una mayor respuesta inmunitaria ^10^. 

Lo resultados falsos negativos pueden estar relacionados con etapas tempranas (CE1) o tardías (CE5) de la enfermedad. El ensayo de inmunotransferencia (Western blot) se utiliza como prueba confirmatoria debido a su mayor sensibilidad y especificidad; se informa, además, que los títulos de anticuerpos disminuyen después del tratamiento quirúrgico, no pudiendo discriminar entre infecciones activas y pasadas ^19^. En nuestro estudio el 76,9% (10/13) de los pacientes tuvieron serología positiva para Western blot de EQ. 

El tratamiento de elección de la EQ es quirúrgico, y la exéresis completa y sin fugas del quiste conlleva a la curación ^10^. En caso de quistes inextirpables o menores de 5 cm, los derivados benzomidazólicos como el albendazol, han mostrado ser efectivos como terapia alternativa o complementaria a la cirugía, mostrando una regresión significativa del tamaño del quiste y alivio de la sintomatología ^10^ ^,^ ^12^. En nuestro estudio se encontró un mayor porcentaje con tratamiento quirúrgico en ambas afectaciones. Los pacientes fueron tratados con albendazol a dosis de 7,5 mg/kg/dosis cada 12 h vía oral (máximo 400 mg cada 12 h). El albendazol es el fármaco más utilizado en niños con EQ, con experiencia limitada en el tratamiento de niños menores de 6 años. Aunque en el pasado albendazol se usó 14 días al mes durante 3 meses, actualmente se indica de 3 a 6 meses consecutivos ^20^; sin embargo, hay pocos estudios disponibles sobre la indicación del tratamiento y la duración de los medicamentos en niños. 

Pocos casos presentan recurrencia después del tratamiento recibido, por ello es importante la monitorización con imágenes cada 3 meses (ecografía o tomografía) por un mínimo de 3 años ^10^; asimismo, se recomienda hacer un tamizaje a los familiares del caso para tener un diagnóstico oportuno y precoz de posibles nuevos casos.

En nuestro estudio, la estancia hospitalaria fue corta y representa la evolución posquirúrgica adecuada. Pocos casos presentaron EQ complicado (quiste hepático complicado y colangitis; quiste hepático complicado con más de diez lesiones, quiste hepático más absceso intraabdominal y quiste pulmonar más empiema). No se reportó mortalidad. 

Igualmente, para lograr el control de la enfermedad es necesario un programa activo de vigilancia epidemiológica en zonas endémicas. A medida que mejore el acceso a exámenes imagenológicos y serológicos lograremos un diagnóstico precoz de EQ en niños en etapa asintomática. 

El estudio tiene limitaciones, como la falta de estandarización en la información clínica de los registros médicos, la correcta clasificación y descripción detallada de los quistes, tiempo de terapia con albendazol y los criterios para la indicación de profilaxis antiparasitaria. Dado que el INSN-SB recibe pacientes referidos con estudios previos de diversos centros y laboratorios del país, la información de estos exámenes auxiliares fue incluida para la toma de decisión quirúrgica en cada caso.

En conclusión, la EQ fue más frecuente en niños en edad escolar y el hígado fue el órgano más afectado, el tratamiento quirúrgico se empleó en la mayoría de los pacientes, no se reportaron eventos fatales.
